# Integrated single-cell and spatial transcriptomics reveals heterogeneity of fibroblast and pivotal genes in psoriasis

**DOI:** 10.1038/s41598-023-44346-6

**Published:** 2023-10-10

**Authors:** Cong-cong He, Tian-cong Song, Rui-qun Qi, Xing-Hua Gao

**Affiliations:** 1 Department of Dermatology, The First Hospital of China Medical University and Key Laboratory of Immunodermatology, Ministry of Health and Ministry of Education, No.155 Nanjing Bei Street, Heping District, Shenyang, 110001 Liaoning People’s Republic of China; 2 Department of Nuclear Medicine, Shengjing Hospital of China Medical University, Shenyang, 110004 People’s Republic of China

**Keywords:** Cell biology, Immunology

## Abstract

Psoriasis, which is one of the most common skin diseases, involves an array of complex immune constituents including T cells, dendritic cells and monocytes. Particularly, the cytokine IL17A, primarily generated by TH17 cells, assumes a crucial function in the etiology of psoriasis. In this study, a comprehensive investigation utilizing bulk RNA analysis, single-cell RNA sequencing, and spatial transcriptomics was employed to elucidate the underlying mechanisms of psoriasis. Our study revealed that there is an overlap between the genes that are differentially expressed in psoriasis patients receiving three anti-IL17A monoclonal antibody drugs and the genes that are differentially expressed in lesion versus non-lesion samples in these patients. Further analysis using single-cell and spatial data from psoriasis samples confirmed the expression of hub genes that had low expressions in psoriasis tissue but were up-regulated after anti-IL17A treatments. These genes were found to be associated with the treatment effects of brodalumab and methotrexate, but not adalimumab, etanercept, and ustekinumab. Additionally, these genes were predominantly expressed in fibroblasts. In our study, fibroblasts were categorized into five clusters. Notably, hub genes exhibited predominant expression in cluster 3 fibroblasts, which were primarily engaged in the regulation of the extracellular matrix and were predominantly located in the reticular dermis. Subsequent analysis unveiled that cluster 3 fibroblasts also established communication with epithelial cells and monocytes via the ANGPTL-SDC4 ligand-receptor configuration, and their regulation was governed by the transcription factor TWIST1. Conversely, cluster 4 fibroblasts, responsible for vascular endothelial regulation, were predominantly distributed in the papillary dermis. Cluster 4 predominantly engaged in interactions with endothelial cells via MDK signals and was governed by the distinctive transcription factor, ERG. By means of an integrated analysis encompassing bulk transcriptomics, single-cell RNA sequencing, and spatial transcriptomics, we have discerned genes and clusters of fibroblasts that potentially contribute to the pathogenesis of psoriasis.

## Introduction

Psoriasis, a chronic and multifactorial inflammatory ailment, primarily impacts the skin, as well as the joints, cardiovascular system, and other organs^1,2^. Notably, psoriasis is characterized by excessive proliferation and expedited differentiation. T cells and dendritic cells are the primary immune cells implicated in the pathogenesis of psoriasis. Specifically, the release of IL23 and IL12 by dendritic cells stimulates T cells to generate cytokines such as IL17, TNFA, and IL22, which subsequently facilitate the excessive proliferation of keratinocytes^3^.

Fibroblasts play a crucial role in preserving the equilibrium of adjacent cells and orchestrating inflammatory reactions, rendering them essential for tissue development, differentiation, and restoration. Under chronic inflammatory conditions, fibroblasts can be activated and exhibit enhanced production of ECM proteins, including type I collagen, tenascin C, SPARC(secreted protein acidic and cysteine-rich), and expression of splicing variants of the additional domain A fibronectin (EDA FN)^4^. Activation is mediated by TGFB, ECM proteases and chemokines^5^. In the dermis, fibroblasts primarily function to synthesize collagen and extracellular matrix. According to reports, soluble factors derived from fibroblasts have an impact on the proliferation and differentiation of keratinocytes, and fibronectin production by fibroblasts is increased in cases of psoriasis^6–8^. In an in vitro skin model, it has been observed that psoriatic fibroblasts stimulate excessive proliferation of normal keratinocytes^9,10^. These findings indicate the involvement of fibroblasts in psoriasis, and further investigation into the specific functions they perform is warranted.

With use of bioinformatic analysis, we successfully identified differentially expressed hub genes in psoriatic lesions and after three anti-IL17A drug treatments. Interestingly, these hub genes were mainly expressed in fibroblasts. As a result of these findings, we further analyzed fibroblast clusters and identified some of their notable functions and regulatory factors.

## Results

### Identified hub genes in psoriasis by Bulk RNA analysis

**Figure 1:**
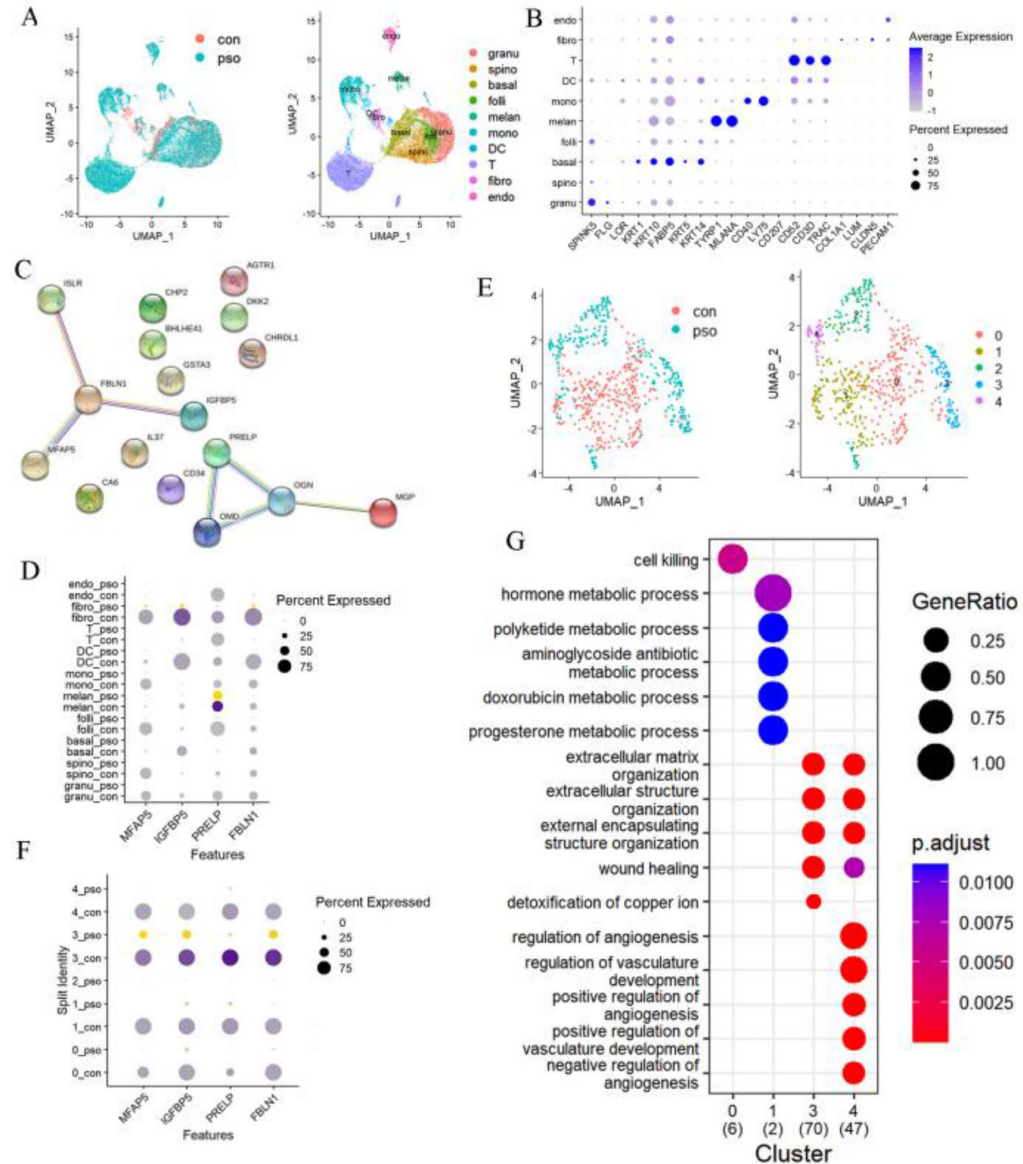
Single cell data of psoriasis (**A**) cells were divided to 10 clusters via umap dimensionality reduction algorithm in healthy and psoriasis tissues (**B**) expression of marker genes in each cluster (**C**) protein–protein network of hub genes low expressed in psoriasis tissue and up-regulated after ANTI-IL17A treatments (**D**) expression of hub genes in each cluster (**E**) subclusters of fibroblasts (**F**) expression of hub genes in fibroblasts (**G**) biological process of each cluster of fibroblast involved.

Bulk RNA transcriptomics of whole skin in psoriasis datasets were analysed. Results of differentially expressed genes(DEGs) as identified following 2 weeks of secukinumab or ixekinumab or 4 weeks of brodalumab treatments showed that 93 genes were down-regulated and 29 up-regulated as compared with their respective baseline levels (Fig. s1a,b, Table s1). To examine the temporally dependent changes in these expressions, determinations of DEGs in response to 6 and 8 weeks of secukinumab and to 12 weeks of brodalumab revealed that 90 down-regulated and 26 up-regulated genes remained. Accordingly, there is little difference in the temporally dependent expressions of these genes following treatments. We also identified DEGs present between lesion versus non-lesion tissue samples of psoriasis patients in the GSE137218 and GSE117468 databases and found 87 genes that were highly expressed in lesion samples and down-regulated after treatments, along with 17 genes showing low expressions in lesion samples and up-regulated after treatments. To further assess the function of these DEGs, gene ontology analysis was conducted. For the 87 genes showing high expression in lesion samples and down-regulated after treatment, their molecular functions were mainly involved with “cell division”, “cell proliferation” and “mitotic nuclear division” (Fig. s1c,d). Unfortunately, the number of DEGs showing low expressions in lesion samples and up-regulated after treatments were too few to enable any clear detection of their functions. With regard to their molecular functions, the structural constituents of the extracellular matrix (ECM) showed the greatest enrichment (Fig. s1e). STRING database was used to construct protein-protein interaction networks and then, with the use of cytoscape, key modules and hub genes were constructed. There was a cluster in the genes highly expressed in lesion samples and down-regulated after treatments, conforming to 27 edges and 351 nodes. Results from calculations of hub genes using cytohubber indicated that ccnb1, ccna2, cdkn3, kpna2, mki67 and cdk1 were hub genes with the highest scores (Fig. s1f,g). For the 17 genes with low expressions in lesion samples and up-regulated after treatment, the PPI network included 9 edges and 9 nodes, with no clusters being involved. The highest ranking genes included FBLN1, IGFBP5, PRELP, OGN, OMD, MFAP5, MGP and ISLR (Fig. 1C). To determine whether these hub genes were related to responses of drugs and assess their specificity as a function of different treatments, patients treated with adalimumab, brodamulab, etanercept and ustekinumab were divided into either responder or non-responder groups using PASI75 as the response criterion (Fig. s1h). Prior to treatments, baseline levels of these hub genes were similar between responders and non-responders. However, after anti-IL17A or methotrexate treatments, most of the hub genes up-regulated in responders

### Hub genes were mainly expressed by fibroblasts

**Figure 2:**
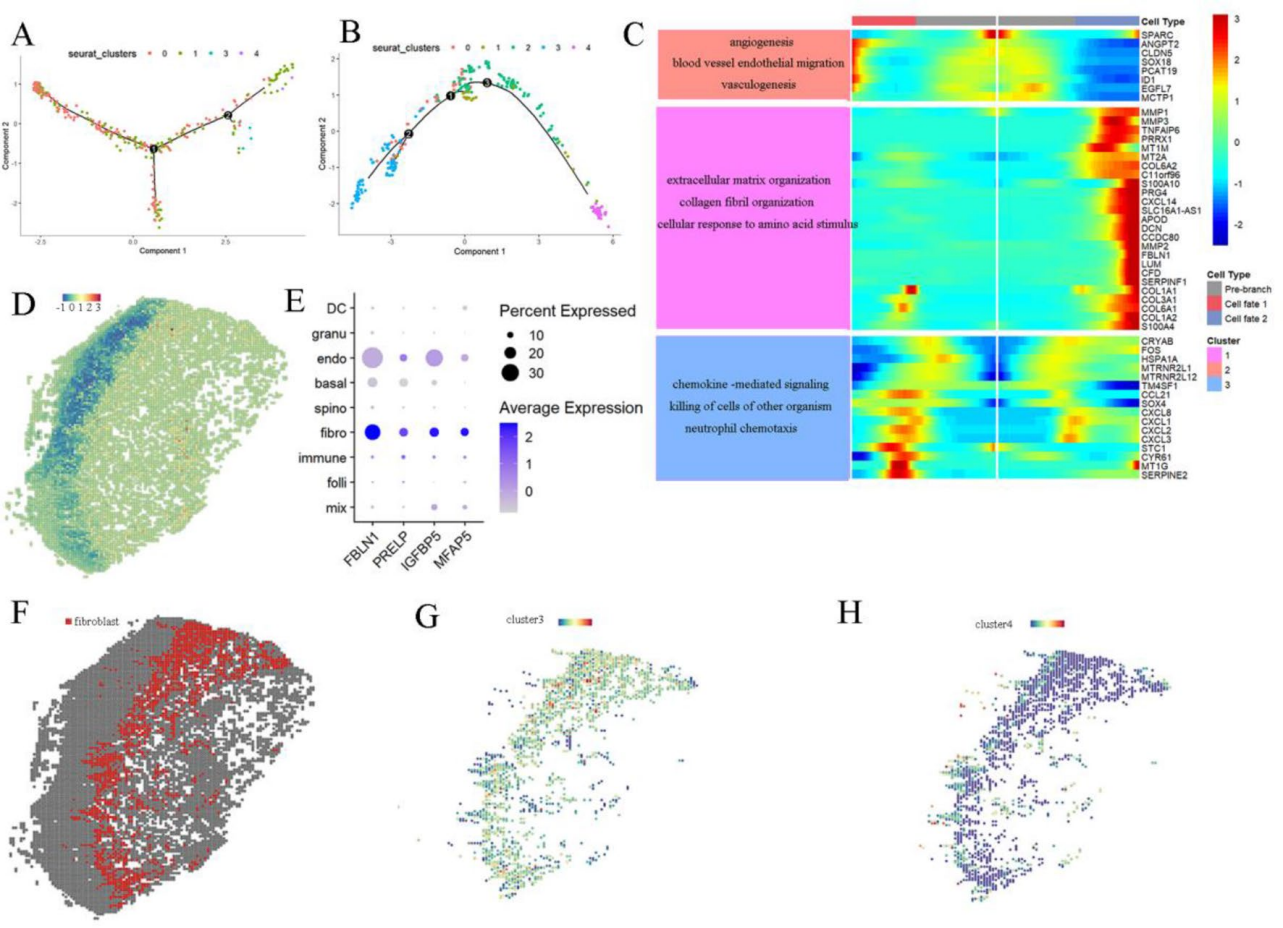
Heterogeneous of fibroblasts in psoriasis (**A**, **B**) pseudotime trajectory of fibroblasts in normal skin and psoriasis skin. (**C**) Branched expression analysis modeling (beam) analysis of genes regulating cell fates, there were two fates of fibroblasts in psoriasis at the first bifurcation. Functions of those genes showed in the left (**D**) expression of PRELP,FBLN1,MFAP5 and IGFBP5 in psoriasis tissue (**E**) expression of hub genes in different clusters in spatial tissue (**f**) distribution of fibroblasts in psoriasis tissue (**G**, **H**) expression model of cluster 3 and 4 fibroblasts identified in single-cell data in spatial.

13 psoriatic lesion samples and 5 healthy samples were constructed as seperate seurat objects and merged. The harmony function was used to remove batch effects. Then 25,455 cells remained and divided into 10 clusters, with unique marker genes for each group (Fig. 1A,B, Fig. s1i). As shown in Fig. 1, expressions of OGN, OMD, MGP and ISLR were all too low to be detected, while other genes were mainly expressed in fibroblasts (Fig. 1D). Consistent with results as obtained from bulk RNA, these hub genes were highly expressed in normal skin, while hub genes were highly expressed in psoriasis and down regulated after treatments in epithelial cells. To further evaluate specific functions of these hub genes showing low expressions in psoriasis and the role of fibroblasts in psoriasis, sub-culsters of fibroblasts were identified (Fig. 1E). Interestingly, fibroblasts in normal skin mainly belonged to clusters 0 and 1, while those in psoriatic skin were mainly in clusters 2, 3 and 4(Fig. 1F). Marker genes in each subcluster are shown in Fig. s2b. In the study of Sole et al., skin fibroblasts were classified into four types, consisting of secretory-reticular, pro-inflammatory, secretory-papillary and mesenchymal. In this study, we identified fibroblasts by taking advantage of this Sole et al. classification (Fig. s2f)^11^. Based on this criterion, fibroblasts in clusters 2 and 4 were classified as secretory-papillary, while those in cluster 3 had the features of both secretory-reticular and mesenchymal. Following a functional analysis of each sub-cluster (Fig. 1G), we found that cluster 0 showed the highest heterogeneity and participated in responses related to interferon gamma, skin development and leukocyte activation. Clusters 1, 2, 3 and 4 were all involved in the regulation of angiogenesis and epithelial migration. Notably, cluster 3 was more enriched in the regulation of extracellular matrix while cluster 4 in the regulation of angiogenesis. The Kegg term, GO terms of “CC”, and “MF” are contained in Fig. s2c-e. Expressions of the ECM component in these clusters were also identified (Fig. s2g) In fact, many components of the ECM, including collagens COL1A1, COL1A2, COL3A1, COL12A1, as well as elastin (ELN), fibronectin (FN1), and fibrillin (FBN1) were all highly expressed in cluster 3, along with a number of other factors involved in matrix assembly, like MFAP4, MFAP5, SFRP2, lysyl oxidase (LOX) and FAP. In contrast, factors involved in matrix remodeling, such as matrix metallopeptidases (MMP2, MMP14), TIMP metallopeptidase inhibitors (TIMP1, TIMP2, TIMP3) and annexin A2 (ANXA2), were not specifically expressed in cluster 3. We performed a trajectory analysis by employing the marker genes in each fibroblasts cluster. In normal skin, fibroblasts clusters 0 and 1 comprised the main fractions and were dispersed in a differentiation trajectory (Fig. 2A), while in psoriatic skin, fibroblasts clusters 3 and 4 were at the terminal portions of differentiation and trajected in different directions (Fig. 2B). These results suggested that fibroblasts clusters 3 and 4 were the main types in psoriasis and exerted distinct functions within this condition. Next, an analysis of gene regulated differentiation was selected (Fig. 2C), with the findings that genes modulating the differentiation of cluster 3 were mainly involved in extracellular matrix organization, while those in cluster 4 were more related to angiogenesis. When cell types were marked in psoriasis tissue at spatial levels, as shown in Fig. s3a, their marker genes were the same as those in the single-cell data (Fig. s3b). Expressions of FBLN1, IGFBP5, PRELP and MFAP5 were all shown to be prominent in fibroblasts (Fig. 2D,E), mostly being expressed in fibroblasts and were also prone to exist in deeper layers of the dermis (Fig. 2D). Distribution of fibroblasts in our tissue was showed in Fig. 2F. Five clusters of fibroblasts were identified as based on single-cell data and were also predicted with spatial analysis, findings which were consistent with previous speculations that cluster 3 was a reticular fibroblasts and cluster 4 a papillary fibroblasts (Fig. 2G,H). Cluster 0 was found to be dispersed in the dermis, while clusters 1 and 2 existed in deeper dermal layers (Fig. s3c).

### Cell communications

**Figure 3:**
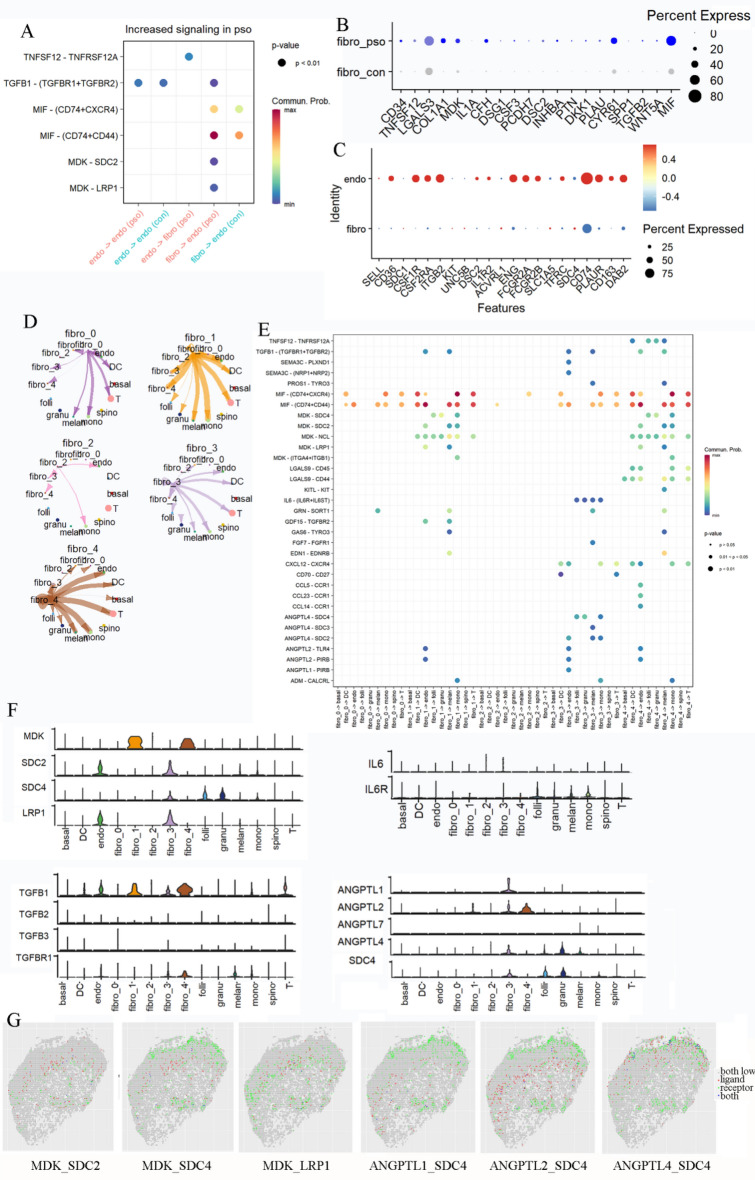
Cell and cell communications in psoriasis (**A**) increased expression of ligand-receptor pair in psoriasis compared with normal tissue (**B**) top 20 ligands in fibroblasts identified by the method of NICHENETR (**C**) top20 receptors on endothelial cells and fibroblasts (**D**) interaction strengths of different clusters of fibroblasts to other cells (**E**) significant ligand-receptor pair of fibroblasts to other cells (**F**) expression of genes in MK, TGFb, IL6 and ANGPTL signals. g. expression of ligands and receptors on spatial transcriptome.

As fibroblasts cluster 4 was mainly involved with the regulation of angiogenesis, as well as with functions involving endothelial cells, we first focused on communications between fibroblasts and endothelial cells. Ligand-receptor configurations in both normal and psoriatic tissue are contained in Fig. s4a, with those showing increased signaling in psoriasis being selected and shown in Fig. 3A. For communications between fibroblasts and endothelial cells, TGFB, MIF and MK represented significant genes associated with communication, which were also computed with the NICHETET method. Several fibroblasts ligands were increased, including TGFB, MIF and MDK. Ligands for fibroblasts and endothelial cells are shown in Fig. 3B and s4c. receptors on fibroblasts and endothelial cells are shown in Fig. 3C. With regard to the 5 types of fibroblasts communicating with other cells, clusters 0 and 2 showed a weak link with other cells, while clusters 1, 3 and 4 displayed a stronger degree of communication with other cells(Fig. 3D,E). For the pairing of receptors and ligands, MIF demonstrated the strongest signal and was present in all cell types. The TGFB signal was enriched in communications between fibroblasts clusters 1, 3 and 4 and endothelial cells and melanocytes, with maximal enrichment in cluster 4. The MK signal, as related to other cells, was shown in fibroblasts clusters 1 and 4. For cluster 3, ANGPTL and IL6 were the unique signals obtained, while expression of genes in MK, TGFB, IL6 and ANGPTL signals were observed in all cells (Fig. 3F). As MDK was mainly expressed in fibroblasts clusters 1 and 4, while their receptors were highly expressed in endothelial cells, we speculated that these clusters regulated vascular endothelial cells through MK signals. We also display MDK and it’s receptors in spatial graphics (Fig. 3G), distribution of MDK was mainly at the junction of epidermis and dermis. ANGPTL1, ANGPTL2 and ANGPTL4 showed a particularly notable expression in cluster 3, and their receptor was highly expressed in follicular keratinocytes, granular keratinocytes and monocytes. In the spatial transcriptome, SDC4 was dispersively expressed, ANGPTL1 and ANGPTL2 were mainly expressed in dermis, and most ANGPTL4 expressed in epidermis or the junction of epidermis and dermis (Fig. 3G). Therefore, cluster 3 fibroblasts, with its high expressions of FBLN1, IGFBP5, PRELP and MFAP5, may communicate with follicular keratinocytes, granular keratinocytes and monocytes through ANGPTL signals. As expressions of TGFB and IL6 were not cluster specific, it seems likely that they might be involved with signals among several different cell types.

### Transcription factors analysis

**Figure 4:**
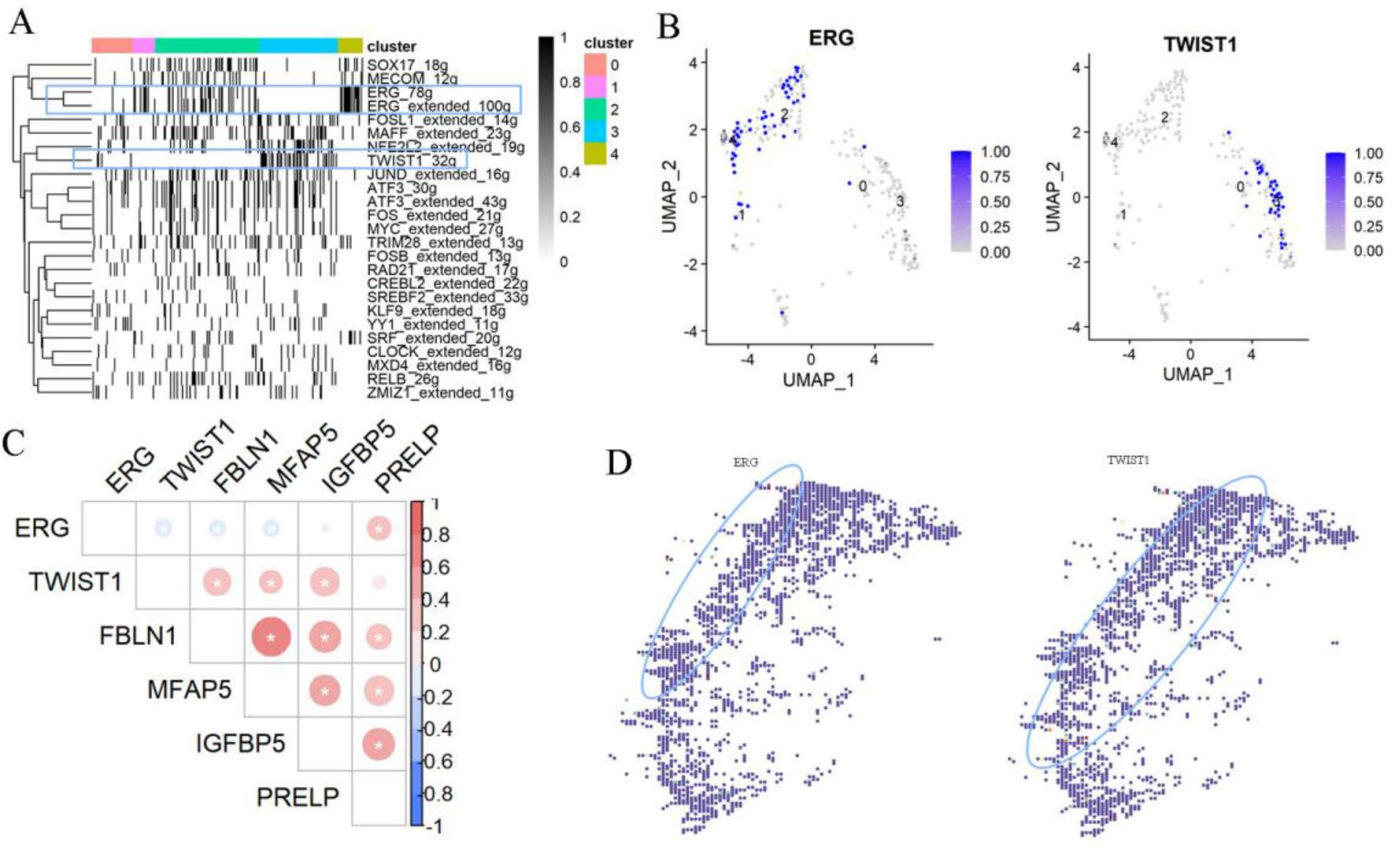
Transcription factors in different fibroblasts clusters (**A**) transcription factor regulons enriched in fibroblasts. “Extended” means the regulons include motifs that have been linked to the TF using motif similarity from SCENIC pipeline, number of genes contained in regulons showed at end (**B**) activity of regulons (TWIST, ERG) in fibroblast (**C**) correlation of genes expressed in fibroblasts (**D**) expression of TWIST1 and ERG in spatial dimension.

SCENIC was used to reveal transcription factors (TFs) of fibroblasts in psoriasis (Fig. 4A). This method integrates co-expression networks and motif based analyses to identify TFs and their regulators. Several TFs were found for cluster 4, with ERG being the most distinct, while for cluster 3, TWIST1 was the most unique TFs (Fig. 4B). There were positive correlations between TWIST1 and PRELP, FBLN1, MFAP5, IGFBP5, which was also specifically expressed in cluster 3, while ERG was negatively correlated with these genes (Fig. 4C). Results of the spatial distributions of TWIST1 and ERG revealed that the expression of TWIST1 was more prone to be located in deeper dermal layers while ERG in superficial regions (Fig. 4D).

## Discussion

Previous bioinformatics analyses of skin psoriasis have indicated a dysregulation in genes such as DEFB4A, S100A7A, and HOTS^12–14^. However, these analyses have also revealed that many markers were concealed in the overall skin transcriptomics analysis. Additionally, single-cell transcriptomics have been employed in the investigation of psoriasis, with particular attention given to TH17 cells, dendritic cells, macrophages, and tissue-resident innate lymphoid cells^15–18^. In Ma's study, which focused on the role of fibroblasts in psoriasis, it was discovered that a specific subset of SFRP2 + fibroblasts produced CCL13, CCL19, and CXCL12 in psoriasis contributing to amplification of the immune network through transition to a pro-inflammatory state^19^. In this study, we conducted an evaluation of alterations in hub genes subsequent to IL17A inhibition therapies in psoriasis. Additionally, we employed single-cell and spatial transcriptomics techniques to examine the functionalities of these hub genes, as identified in bulk RNA data. Furthermore, this study identified five distinct clusters of fibroblasts with diverse functions and distributions, thereby enhancing our comprehension of fibroblasts in the context of psoriasis.

Notably, when comparing differentially expressed genes (DEGs) at various time points subsequent to three anti-IL17A drug treatments, it is intriguing to observe the acquisition of similar DEGs, suggesting that DEGs which changed, did so early in the treatment regimens and sustained their expression for a relatively long duration. Regarding the differentially expressed genes (DEGs) observed between samples of lesion and non-lesion skin, it was observed that the majority of genes exhibiting up-regulation following treatments had low expression levels in lesion tissue samples. Notably, CCNB1, CCNA2, CDKN3, KPNA2, MKI67 and CDK1, identified as hub genes, were found to be associated with proliferation, mitosis, and cell cycle regulation^20–22^. These findings align with the established role of anti-IL17A drugs in psoriasis, which inhibit proliferation. Conversely, the genes displaying down-regulation after treatments were found to be highly expressed in lesion tissue samples. The hub genes, FBLN1, IGFBP5, PRELP, OGN, OMD, MFAP5, MGP and ISLR have received only scant research regarding their function as related to psoriasis. FBLN1 encodes Fibulin-1, a secreted protein related to basement and elastic extracellular matrix fiber, is highly expressed in skin, heart, blood vessels and the lungs^23^, and plays a role in several diseases including respiratory conditions and certain types of carcinoma^24,25^. Insulin-like growth factor (IGF) regulates growth of the epidermis and appears to be regulated by IGF binding protein (IGFBP5)^26^. Expression of IGFBP5 can be reduced by IFN and TNFA as stimulated in fibroblasts, while the complex of IGF-I:IGFBP-5:vitronectin increases protein synthesis and migration of HaCat cells^27^. Therefore, the role of IGFBP5 in psoriasis may be controversial and requires further investigation. PRELP, OGN, OMD and MGP encoding extracellular matrix proteins involved in cell adhesion and bone regulation^28–30^ they also related to effective treatments in response of brodamulab and methotrexate in our study. Methotrexate is a folic acid analogue that is classified as an immune-suppressant and antineoplastic agent and is believed to play a role in anti-inflammation and anti-proliferation at low doses, such as in the treatment of psoriasis and rheumatoid arthritis^31–33^. As those genes show similar expression profiles after brodamulab and methotrexate treatments, it seems likely that they involve equivalent mechanisms in the treatment of psoriasis. In contrast to that of brodamulab and methotrexate, those genes are not related to the treatment effects of adalimumab, etanercept and ustekinumab. The involvement of monoclonal antibodies to TNFA and IL12/IL23 suggests that these latter treatments are associated with different mechanisms. As datasets on secukinumab and ixekizumab treatments lack PASI scores, any relationships on expressions of their hub genes and treatment responses will need to be verified.

In the present study, we focused on fibroblasts as results from data obtained with single-cell and spatial transcriptomics indicated that the hub genes identified were mainly expressed in fibroblasts. Fibroblasts are mesenchymal cells that deposit collagen and elastic fibers of the ECM in connective tissue^34^. In psoriasis, fibroblasts produce soluble factors, such as keratinocyte growth factor (KGF), to induce proliferation of keratinocytes^35^. Natsumi^36^ reported that laminin-511, which is produced by fibroblasts, induces proliferation and inhibits apoptosis in HaCat cells and laminins enhances neutrophil recruitment and phagocytic ability of dendritic cells. The fibroblasts in our study were divided into 5 clusters, among which, clusters 3 and 4 were fibroblasts that mainly exist in psoriasis tissue and share distinct functions and distributions. Regulation of the ECM represents the main function of cluster 3, which is prone to be distributed in deeper dermal layers in psoriasis tissue. Cluster 4 fibroblasts, which closer to the epidermal layer, are mainly involved in vascular regulation. Although their distribution has been verified in a previous study^11^, their function requires further investigation.

Severe angiogenesis is a characteristic of psoriasis pathology, which then results in inflammatory responses. Fibroblasts regulate endothelial cells via various mechanisms and, by controlling production of the extracellular matrix and growth factors, fibroblasts play a role in growth and stabilization of the vasculature^37^. However, the crosstalk between fibroblast and endothelial cells in psoriasis is rarely investigated. MDK (Midkine)-SDC has been speculated to link fibroblasts with endothelial cells, with cluster 4 fibroblasts playing a role in the regulation of vascular function. MDK is a heparin-binding growth factor implicated in several physiological processes including development, reproduction, repair and angiogenesis^38,39^. As MDK is a stimulator of collagen and glycosaminoglycan synthesis^40^, it seems reasonable to infer that MDK regulates vascular endothelial function through a stimulus-induced production of collagen in fibroblasts. ANGPTL4 can regulate several aspects of fibroblast functions, such as orienting the configuration of fibrous matrices, activation of cancer-associated fibroblasts in tumors and co-localization with fibroblast secreted protein-1 and α-smooth muscle actin^41–43^. SDC4 is a member of the SDC family, which is involved in signal transduction processes across cell membranes and plays a role in cell adhesion, proliferation and migration^44^. TWIST1 can regulate fibrosis within the kidney, lung and other tissues, and is also involved in epithelial-mesenchymal transition in a variety of diseases^45,46^. It has been reported that TWIST1 promotes colony formation of mouse embryo fibroblasts^47^, but the specific function of TWIST1 in fibroblasts is not clear. Our current results indicate that TWIST1 can serve as a special transcription regulator of cluster 3 fibroblasts, however any further relationships that may exist will require further verification. The transcriptional regulator, ERG, has been identified as a key modulator of endothelial function that not only regulates angiogenesis and vascular stability, but also promotes homeostasis of the vascular endothelium^48^. ERG is a key transcription regulator of cluster 4 fibroblasts, which have been identified as comprising a cluster of fibroblasts involved in vascular function in psoriasis.

In conclusion, our study analyzed the classification and distribution of fibroblasts in psoriasis in detail, which help to further our understanding of fibroblasts and the pathogenesis of psoriasis. Several molecules found in our study may be potential targets for treating psoriasis.

## Methods

### Bulk RNA datasets

Datasets from the Gene Expression Omnibus (GEO, https://www.ncbi.nlm.nih.gov/geo/) of The National Center for Biotechnology Information (NCBI) were reviewed. GEO2R, an online analysis tool built in the GEO website, was used to analyze raw data to identify DEGs, with a *P* value of < 0.05 and |Foldchange| of > 1 used as cut-off values. In the GSE137218 dataset, 14 patients received subcutaneous secukinumab 300 mg, for 12 weeks. Biopsies of whole skin were obtained on days 0, 4, 14, 42 and 84 during the treatment period. In the GSE31652 dataset, 8 patients received subcutaneous ixekizumab 150 mg, and biopsies obtained in weeks 0 and 2. In the GSE117468 dataset, 46 patients received 140 mg and 41 received 210 mg of subcutaneous brodalumab and biopsies obtained in weeks 0, 4 and 12. DEGs was identified in week 0 and other point of time for the three datasets. DEGs as obtained in lesion versus non-lesion tissue were also identified in the GSE137218 and GSE117468 datasets. In the GSE85034 dataset, 15 patients received methotrexate (7.5 mg per week) and 15 received adalimumab (80 mg, sc). DEGs were analyzed at week 16 after treatments for both arms. In the GSE117239 dataset, 50 patients received ustekinumab (45 or 90 mg) and 33 received etanercept (50 mg) with analysis of DEGs identification performed at 12 weeks after treatments. Volcano diagrams of DEGs were drawn using the sanger box, an online tool. The DAVID knowledgebase (https://david.ncifcrf.gov/), an online gene functional annotation tool, was used to analyze the functions and pathway enrichments of obtained DEGs and marker genes^49^. The *P*-value was calculated using the Fisher exact test with a *P*-value of < 0.05 regarded as being statistically significant. To analyze interactions among proteins of interest, the STRING platform, an online tool for the structural and functional analysis of protein interactions was used. Subsequently, Cytoscape software 3.6.1 (https://cytoscape.org) and built-in app Molecular Complex Detection (MCODE) were used to identify core modules in these proteins and cytoHubba was used to calculate hub genes based on the overlapping results as obtained with MCC (Maximal Clique Centrality) topological analysis methods.

### Analysis of scRNA datasets

GSE151177 was downloaded from the GEO database, and included 13 human psoriasis skin lesion samples and skin samples from 5 healthy volunteers as controls. The Seurat package was used in R version4.1. In detail, 13 seurat object were construct using “CreateSeuratObject” function, genes expressed by less 10 cells were eliminated. Also, cells with < 200 unique genes and with very high mitochondrial(> 30% mitochondrial reads) were filtered out. After merging those 18 objects, harmony was used to remove batch effects, then 2,000 top variable genes were isolated to obtain clusters. The Uniform Manifold Approximation and Projection (UMAP) algorithm was applied for dimensionality reduction with dimension 1:15. Marker genes of each cluster mainly included those as referenced in a previous study^50^ and in the online database-CellMarker.

### Trajectory analysis

The Monocle2 package was used to analyze the pseudotime trajectory of fibroblasts in single-cell datasets, with genes differentially expressed in subclusters being used. This algorithm is a machine learning technique used to acquire a parsimonious principal graph, as achieved by reducing high-dimensional expression profiles to low-dimensional spaces. Cells with the same differentiation fate existed in the same branch and branched expression analysis modeling (BEAM) was used to identify genes regulating the fate of cells.

### Cell communications

Communications among cells from psoriasis and control skin samples in single-cell datasets were analyzed using the cellchat package^51^. With this program it is possible to predict major signaling inputs and outputs of cells, as well as the means through which these cells and signals coordinate their functions with use of network analysis and pattern recOGNition approaches. The Nichenet package were also used to identify ligands, receptors and related targets in cell communications.

### Analysis of transcription factors

GENIE3 was used to infer the co-expression network, followed by RcisTarget for the analysis of transcription factor binding motifs. Finally, AUCell was used to identify cells with active gene sets (gene-network) in the scRNA-seq data.

### Spatial transcriptome methods

Fresh tissues were dissected and rinsed in the pre-cooled PBS (DEPC treated) on the ice to remove unwanted debris or fluids, then cleaned using sterile gauze to absorb excess liquid on the tissue to avoid ice formation. The dissections were then embedded in the pre-cooled cryomold on the dry ice using Tissue-Tek® O.C.T. Compound (SAKURA, 4583). Embedded tissues were sealed and stored in -80℃ until cryosection. Cryostat chamber was pre-cooled and cryosections were cut at a thickness of approximately 10 μm. The most qualified slice was ready for subsequent experiments and closely neighbor slices were used to conduct hematoxylin and eosin (H&E) staining to confirm the characteristic of target tissue. STOmics library preparation and sequencing was conduct by standard process of BGI. The raw spatial expression matrix was convoluted into pseudo-spots (bin 50) of 25 um diameters, with one spot regarded as a cell. Those spots was then clustered as that of single cells. Expressions of hub genes were scored together as based on their functions with use of “AddModulScore” and then exhibited on tissues by “SpatialFeaturePlot”. Expression of ligands and receptors was used SpaGene R software.

### Supplementary Information

Supplementary Figures.

Supplementary Table S1.

## Data Availability

Accession number of raw data is “PRJNA1011360” in SRA database.
