# Supplementary material for: Integrated single-cell and spatial transcriptomics reveals heterogeneity of fibroblast and pivotal genes in psoriasis
Source: Sci Rep. 2023 Oct 10;13:17134. doi: 10.1038/s41598-023-44346-6 (PMC10564713; doi:10.1038/s41598-023-44346-6)

Figure.S1:Bulk RNA data of three psoriasis datasets.a.differentially expressed genes(DEGs) in the three datasets,red points means genes up-regulated after treatments,green points means genes down-regulated after treatments,P value < 0.05,|Foldchange | > 1;b.veen diagram of the DEGs in the three datasets,the left means down-regulated after treatments and high expression in lesional tissues,the right means up-regulated after treatments and low expression in lesional tissues;c-d.GO and KEGG analysis of DEGs down-regulated after treatments and high expression in lesional tissues;e.GO analysis of DEGs up-regulated after treatments and low expression in lesional tissues;f-g.protein-protein interaction(PPI) of DEGs down-regulated after treatments and high expression in lesional tissues and a model.h.expression of hub genes after treatment by adalimumab,brodamulab,etanercept,methotrexate and ustekinumab. I.Feature plots of marker genes in each cluster.

Figure.S2:a.expression of genes high expression in psoriasis tissue and down-regulated after treatments in single-cell clusters.b.top 5 marker genes in each cluster of fibroblast.c-e.KEGG,cell component and molecular function term of each cluster of fibroblast.f.expression of genes been identified to represents different type of fibroblast in Solé et al. study. g. Extracellular matrix related genes expressed in clusters of fibroblast.

Figure.S3:a.spatial distribution of cell types in psoriasis tissues.b.expression of marker genes in psoriasis tissue.c.expression model of cluster0,1 and 2 fibroblast identified in single-cell data in spatial.

Figure.S4:a.strength of ligands and receptors pair in normal and psoriasis group.b.relative information flow in the two group.c.interaction potential of ligand and receptor in psoriasis compared to normal skin.

Table.S1: list of genes up or down regulated after treatments by anti-IL17A drugs.

Figure.S1：


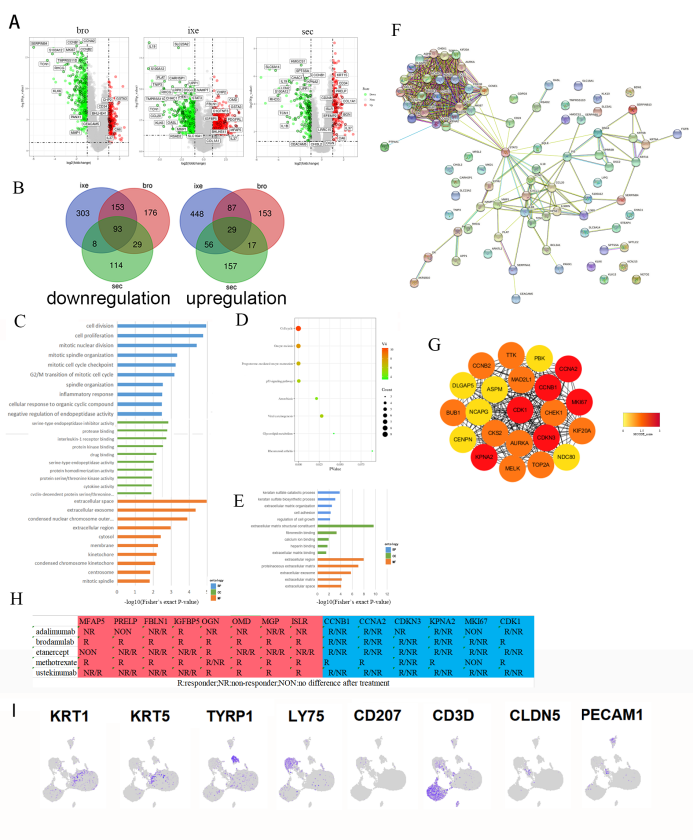


Figure.S2：


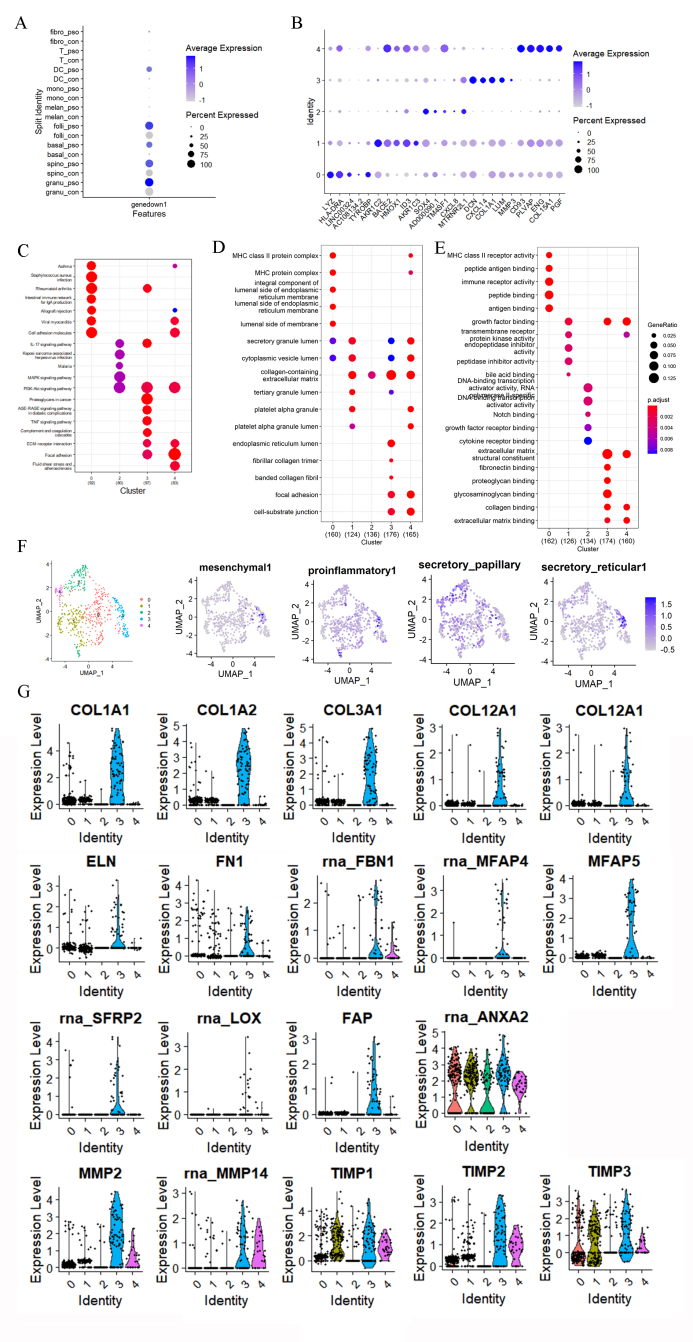


FIGURE.S3:


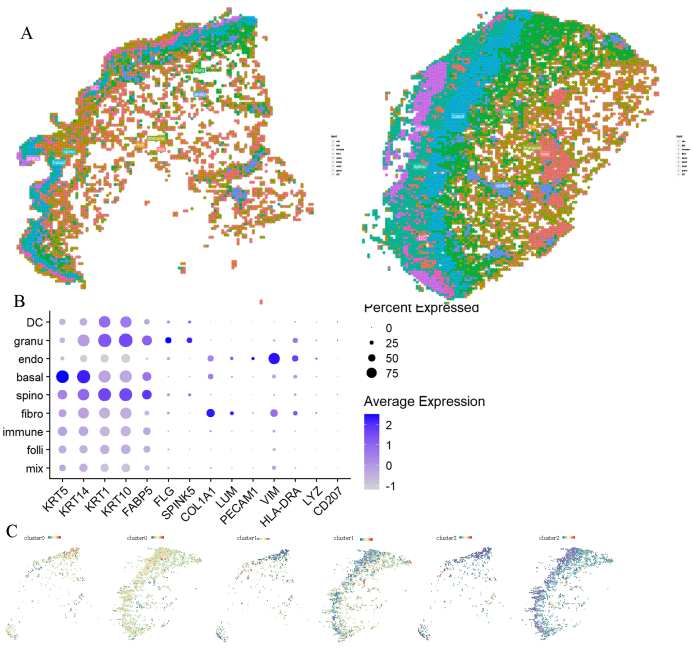


FIGURE.S4:


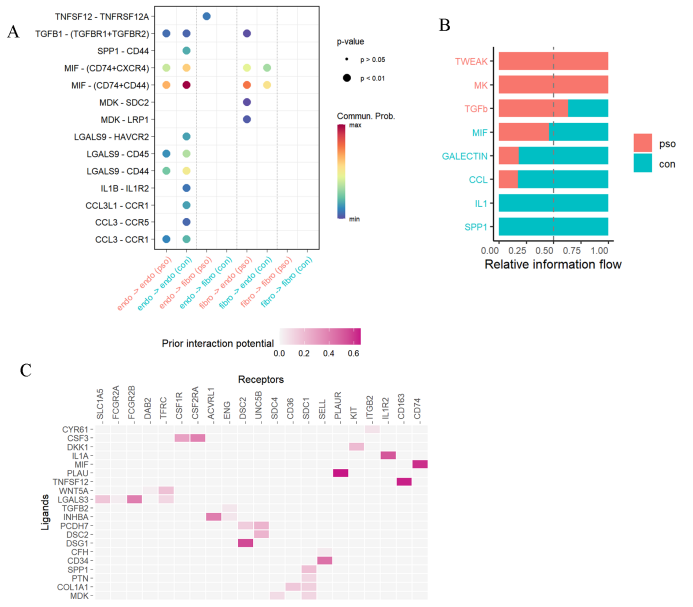

Supplement: Supplementary file 1 — Supplementary Figures. [file 41598_2023_44346_MOESM1_ESM.docx]
